# The neural basis underlying the association between parents’ socioeconomic status and depressive symptoms among college students

**DOI:** 10.3389/fpsyg.2024.1464273

**Published:** 2024-11-25

**Authors:** Yao Xiao, Xinting Jiang, Yuan Li, Yu Mao, Duyi Zhou

**Affiliations:** ^1^College of Teacher Education, Southwest University, Chongqing, China; ^2^School of Educational Sciences, Chongqing Normal University, Chongqing, China; ^3^College of Artificial Intelligence, Southwest University, Chongqing, China

**Keywords:** socioeconomic status, depressive symptoms, default mode network, ventral attention network, functional connectivity pattern

## Abstract

**Objective:**

Depression is increasingly prevalent among adolescents, with parents’ socioeconomic status (SES) serving as significant predictors. Understanding the link between parents’ SES and college students’ depressive symptoms is of paramount concern. However, the neural basis linking the association between parents’ SES and students’ depressive symptoms still remains to be explored. In order to address this issue, this study aims to investigate the relationship between parents’ SES and students’ depressive symptoms, and the role of brain functional connectivity (FC) pattern in this relationship.

**Methods:**

In this study, a total of 363 college students without a history of mental or neurological disorders underwent depressive symptoms assessment and resting-state functional magnetic resonance imaging scans. We used a connectome-based predictive modeling (CPM) approach to identify neural biomarkers of depressive symptoms.

**Results:**

The results indicate that there is a negative correlation between parents’ SES and students’ depression tendencies (Father’s education level and SDS: r  = −0.119, *p* < 0.05; Mother’s education level and SDS: r  = −0.117, *p* < 0.05), suggesting that students whose parents have a higher educational level are less likely to suffer from depression. Furthermore, a FC pattern that can significantly predict depressive symptoms outside of the body was identified (r  = 0.13, *p* < 0.005), with most of the FCs belonging to the default mode network (DMN) and ventral attention network (VAN). Additionally, the FC pattern associated with depressive symptoms mediate the relationship between parents’ SES and depressive symptoms.

**Conclusion:**

Therefore, we believe that improving the education levels of parents may have a practical effect in reducing depressive symptoms among adolescents.

## 1 Introduction

Socioeconomic status (SES), a psychological and social construct, is measured by criteria that evaluate economic resources and typically functions as a framework for societal stratification (McLoyd, 1998; Farah, 2017). SES comprises a complex, multidimensional structure. There is a strong association between diverse variations in individual brain connectivity and the three factors of SES: parental education level, neighborhood disadvantage, and household income relative to needs. Research focusing on the unique effects of each aspect of SES indicates that the connection with parents’ educational attainment is the strongest, even when considering factors such as family financial needs and neighborhood challenges (Sripada et al., 2022). Generally, individuals with a higher socioeconomic status have an abundance of resources and benefits at their disposal (Akinola and Mendes, 2014). In contrast, those from lower social and economic positions often face challenges such as limited resources and financial struggles (Tan et al., 2020). Parents’ SES significantly influences an individual’s life from childhood to adulthood, particularly affecting those from low-income backgrounds. In such circumstances, health, psychological well-being, and cognitive and emotional development are negatively impacted throughout the life course. The role of parents’ SES in individual mental health, cognitive function, and brain development has garnered widespread attention.

Mental health conditions can vary depending on parents’ SES. A lower economic status correlates with an increased likelihood of health issues throughout a lifetime, including depression (Goodman, 1999; Kivimäki et al., 2020), compared to those from affluent backgrounds with more resources. Depression, characterized by affective disorders, presents with depressive states, feelings of letdown, and despair (Gotlib and Hammen, 2009), and frequently occurs as a mental health issue in teenagers (Vos et al., 2015). Parental education level, a critical parents’ SES indicator (Bradley and Corwyn., 2002), predicts a more advantageous home environment for children, particularly concerning economics, resources, and prospects, with higher-educated parents typically providing these advantages (Reiss et al., 2019). Essentially, as parents’ education levels rise, adolescents’ socioeconomic standing increases, leading to a reduced likelihood of them suffering from depression. As an example, the effect of parental learning on teenage depression originates from its impact on parental thought processes and behavior. Parents with higher education levels often adopt more beneficial and logical parenting approaches to foster mental health in their teenagers, thereby decreasing depression risks (Carr and Pike., 2012). When children experience mental health problems, their mothers are not only willing to play the role of caregiver but also to play the role of co-patient/client (Giannouli and Stoyanova, 2014). families with lower socioeconomic status may experience higher levels of maternal depression, meanwhile, maternal depression and sensitivity have an impact on negative mood and behavioral disorders in offspring (Bouvette-Turcot et al., 2020), and offspring of depressed mothers have a higher risk of depression (Pawlby et al., 2009). Wetherall et al. (2019) conducted a comprehensive analysis of psychological health, emphasizing 29 studies from various countries like the USA, Germany, South Africa, UK, and China, which had earlier explored the link between parents’ SES and depressive indicators. The research focused on the relationship between socioeconomic standing and symptoms of depression, revealing that individuals with higher socioeconomic status are less likely to experience depression (Wetherall et al., 2019). In sum, as an important component of SES, parent’ s education levels significantly predicts progeny depression (Ritsher et al., 2001).

Parents’ SES is a known predictive factor of individual differences in brain structure and functional systems throughout the entire lifecycle (Chajes et al., 2022). With the onset of the teenage years, adolescents enter their second critical phase of brain development, especially in areas associated with advanced cognitive functions and purpose-driven actions (Steinberg et al., 2018). Research has indicated that parents’ SES could affect brain-related aspects of advanced cognitive functions in teenagers, such as the ability to control thoughts (Lawson and Hook, 2018). The development of the prefrontal cortex renders cognitive regulation vulnerable to environmental factors. Hindered conditions, like lower parents’ SES, can hinder mental control in adolescents (Brieant et al., 2021; Lambert et al., 2016). Specifically, the extent of a parent’s education is closely linked to an expanded region in the anterior cingulate cortex and inferior frontal gyrus, which are essential for executive skills and cognitive control (Brito and Noble, 2018; Noble et al., 2015). Current research underlines the critical role of cognitive control in managing emotional responses and addressing emotional incidents (Straub et al., 2022). Previous study found that reduced cognitive control, coupled with intense short-term thinking, was a predictor of a rise in adverse feelings, including depression (Pe et al., 2013). Conversely, better working memory updating predicts that reassessment is more efficient in reducing negative emotions (Hendricks and Buchanan, 2016). Research also indicates a direct link between childhood cognitive control deficiencies and a subsequent increase in depressive indicators (Kertz et al., 2016). Higher parents’ SES (i.e., higher income or a higher level of maternal education) was reported to be associated with lower connectivity within default mode network (DMN; Gao et al., 2015). Meanwhile, abnormalities in functional connectivity within the DMN have also been established as neural markers of depression (Yan et al., 2019). Moreover, previous study also suggesting that parents’ SES moderates the association between income and connectivity within the dorsal and ventral attention networks (VAN; DeJoseph et al., 2022).

However, numerous studies have focused exclusively on the impact of parents’ SES on depression and its psychological processes from a behavioral perspective (Xue et al., 2021; Zeng and Xu, 2024), and most studies rely on small, non-representative samples (Habersaat et al., 2018; Davidson et al., 2023). Thus, the neurobiological underpinnings of the influence of parents’ SES on depression remain unclear. Consequently, acknowledging the limitations of past research, the objective of this study was to explore the neural basis that explain the connection between partents’ SES and students’ depression. Initially, we employed connectivity-based predictive modeling (CPM) to determine the neurological indicators of depression. CPM is a machine learning technique for creating predictive models that analyze interactions between brain and behavior, using feature-based data with cross-validation (Shen et al., 2017). Subsequently, we examined whether the brain mechanisms underlying depression can explain the link between depressive symptoms and parents’ SES. Furthermore, we hypothesized that functional connectivity related to depression could mediate the relationship between depressive symptoms and parents’ SES. Based on previous studies, we hypothesized that parents’ education levels were robust predictors of students’ depressive symptoms. Morevoer, the FC of DMN may be the underlying neural basis linking parents’ education levels and students’ depressive symptoms.

## 2 Methods

### 2.1 Participants

The study’s behavioral and imaging data were sourced from the SLIM Brain Data Repository at Southwestern University, accessible for research use through the International Data-sharing Initiative (INDI, https://fcon-1000.projects.nitrc.org/). Participants learned about the recruitment channels through flyers, online advertisements, and face-to-face campus interactions. Those who were fluent in Chinese and were first-year or second-year students were eligible to participate in SLIM. Participants gave their written consent before the experiment began and received payment following the study. The research received its ethical approval from the Ethics Committee of Southwest University (dated March 6, 2023; H23061), and all procedures adhered to the sixth edition of the Declaration of Helsinki. Participants exhibiting significant head movement (average FD power beyond 0.2) during resting-state functional magnetic resonance imaging (fMRI) were excluded from the study. Specifically, A total of 363 participants (mean age 19.99 + 1.25 year; range: 17–27 years; 160 males and 203 females) from the SLIM performed assessment of depression scale and underwent resting-sate fMRI scans, 352 of them (mean age 19.99 + 1.24 year; range: 17–27; 198 females and 154 males) filled out the father’s education level, and 352 of them (mean age 19.98 + 1.22 year; range: 17–27; 197 females and 155 males) filled out the mother’s education level. The project was solely focused on Chinese undergraduates who meet the MRI-related exclusion criteria, have no psychiatric disorders, have not used psychiatric medications within the 3 months prior to scanning, are not pregnant, and have no history of head trauma.

### 2.2 Measures

The Self-Rating Depression Scale (SDS). This self-administered survey comprises 20 questions, categorized into four aspects: affective, physiological disruptions, psychomotor issues, and psychological turmoil associated with depression (Zung, 1965; Lezak, 2004). Each of these items in the SDS was rated on Likert 4 point range from 1 to 4 (rarely, sometimes, often, most of the time). A total score < 50 corresponds to absence of depression, while 50–59, 60–69, and > 70 indicate slight, severer depression, and moderate, respectively (Zung, 1965). The study used Chinese Version of the Self-Rating Depression Scale. With a Cronbach’s alpha of 0.73 for the SDS, this indicated internal consistency in assessing a fundamental aspect of depression.

The parents’ SES measured by household annual income and parents’ education level. Data of household income were collected using discrete variables: (1) annual income < RMB 5,000; (2) annual income RMB 5,000–15,000; (3) annual income RMB 15,001–30,000; (4) annual income RMB 30,001–50,000; (5) annual income RMB 50,001–100,000; (6) annual income > RMB 100,000. The values 1–6 were used in subsequent regression analyzes (Takeuchi et al., 2014). Similarly, Parents’ educational attainment was evaluated using a Likert 5 scale that spans from 1 to 5 points: (1) below primary education; (2) primary education, secondary school; (3) high school; (4) university and (5) above. This study incorporates these variables into its demographic analysis.

### 2.3 fMRI data acquisition and analysis

#### 2.3.1 Image acquisition and preprocessing

During the resting-state MRI scan, participants were to recline, eyes closed, and remain at rest, avoiding any intentional thought while not allowing sleep. The 8-min scan of 242 contiguous whole-brain resting-state functional images was obtained using gradient echo echo-planar-imaging (GRE-EPI) sequences with the following parameters: slices = 32, flip angle = 90, repetition time (TR)/echo time (TE) = 2000/30 ms, thickness/slice gap = 3/1 mm, field of view (FOV) = 220 × 220 mm, and voxel size = 3.4 × 3.4 × 4 mm^3^.

Each piece of resting-state fMRI data was preprocessed using the Data Processing Assistant for Resting-State fMRI (DPARSF_V4.2, http://resting-fmri.sourceforge.net/) on MATLAB 2014a (Math Works, Natick, MA, USA) as the operating system. Participants whose head movement exceeded 2.0 mm in any dimension during the scan were excluded from further analyses. The task entailed these steps: exclusion of initial 10 images, slice-timing correction, realignment, spatial standardization, regression of nuisance signals, data cleaning, spatial smoothing, and band-pass filtering. These preprocessing procedures followed established protocols (Yan et al., 2016).

#### 2.3.2 Functional connectivity estimation

After preliminary data processing, the complete brain functional connectivity (FC) matrix was developed using the Graph Theory Network Analysis (GRETNA) toolbox (Power et al., 2013; Wang et al., 2015) delineated an outline comprising 264 potential functional areas incorporating 14 networks. After excluding 5 networks (auditory, sensory hand, sensory mouth, visual and uncertain network), the present study remained to included 9 networks (frontoparietal, cingulo-opercular, dorsal attention, salience, ventral attention, subcortical, default mode, memory retrieval, and cerebellar network) and 157 nodes. Determined the blood oxygenation-level-specific time frames at each participant’s point, using Pearson correlation coefficients to assess the FC intensity across these node pairs. Using Fisher’s formula, correlation coefficients were transformed into z values (Baltosser and Zar, 1996). Consequently, each participant’s complete connectivity matrix comprised a 157 × 157 dimensional z-score matrix, with every edge incorporated as predictive features in the model.

### 2.4 Connectome-based predictive modeling

We present connectome-based predictive model (CPM) is an empirical approach designed to estimate depression levels in individuals by evaluating whole-brain FC through a K-fold cross-validation method (Shen et al., 2017). Age, sex and mean FD power were used as covariates in CPM algorithm. Initially, Pearson correlation tests were conducted to determine the correlation between every depression score and each edge of the participants’s connectivity matrix. Then, a threshold (here: threshold = 0.005) was employed to eliminate weak connections while retaining strongly correlated edges. The network of positive predictors included connections positively associated with depression levels, unlike the inversely correlated connections that constituted the negative predictive network. To assess the network’s positive robustness for each participant, we summed up their network connections’ strength and fed them into predictive models for depression ratings, presuming linear correlations. Third, we used K-fold (K = 10) cross-validation, create a predictive model using linear regression to correlate each participant’s network strength with their recorded depression scores. During K-fold cross-validation, participants were divided into K equally sized groups. Throughout every cycle, (K-1) cohorts were employed for educational purposes, and the rest for evaluation. Each participant’s predicted scores were obtained after completing all K folds. Pearson’s correlation method was used to evaluate the accuracy of the model in predicting the relationship between predicted and actual outcomes. For enhanced steadiness in the r and p measurements, the cross-validation process was conducted 10 times.

### 2.5 Statistical analysis

#### 2.5.1 Demographic and correlation analysis

All analyses of correlation were performed by SPSS 26.0 software (IBM Corp., Armonk, New York, NY). We used the Spearman correlation method to assess the relationship between parents’ SES and depression.

#### 2.5.2 Mediation analysis

Utilizing SPSS’s PROCESS macro (Preacher and Hayes, 2008), our investigation focused on how resting-state FC influences the effects of parents’ education on depression. Specifically, Model4 in PROCESS macro was used for the analysis. Our initial model utilized the father’s education level as the primary independent factor, FC as the intermediary, and depression as the resulting variable. Utilizing the bootstraps technique, which included 5,000 iterations, the importance of the mediating impact was assessed. If 95% confidence interval (CI) excludes 0, the intermediate effect is significant. In the second model, we performed a mediation analysis with mother’s education level as the outcome variable, maintaining the original formatting for consistency.

## 3 Results

### 3.1 Correlations among variables

Descriptive statistics of behavioral data were presented in Table 1. Our results revealed that college students’ depressive symptoms were negatively correlated with father’s education level (r = −0.119, *p* < 0.05) and mother’s education level (r = −0.117, *p* < 0.05), but not household Annual Income (r = −0.087, *p* > 0.05).

**Table 1:** Means and standard deviations of variables.

	Number	Mean	SD
Household annual income	352	3.44	1.175
Father’s education level	352	3.21	1.079
Mother’s education level	352	2.84	1.167
SDS	363	34.26	8.471

^*^p < 0.05.

### 3.2 CPM results

**Figure 1:**
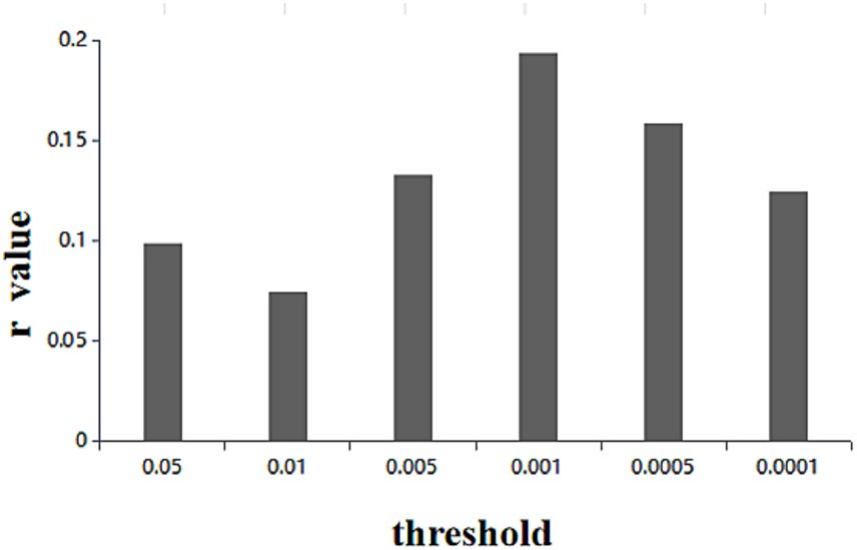
The results of CPM at different threshold values. The x-axis represents the threshold values in the edges selection step and the y-axis represents the correlation coefficient between predict value and actual value.

**Figure 2:**
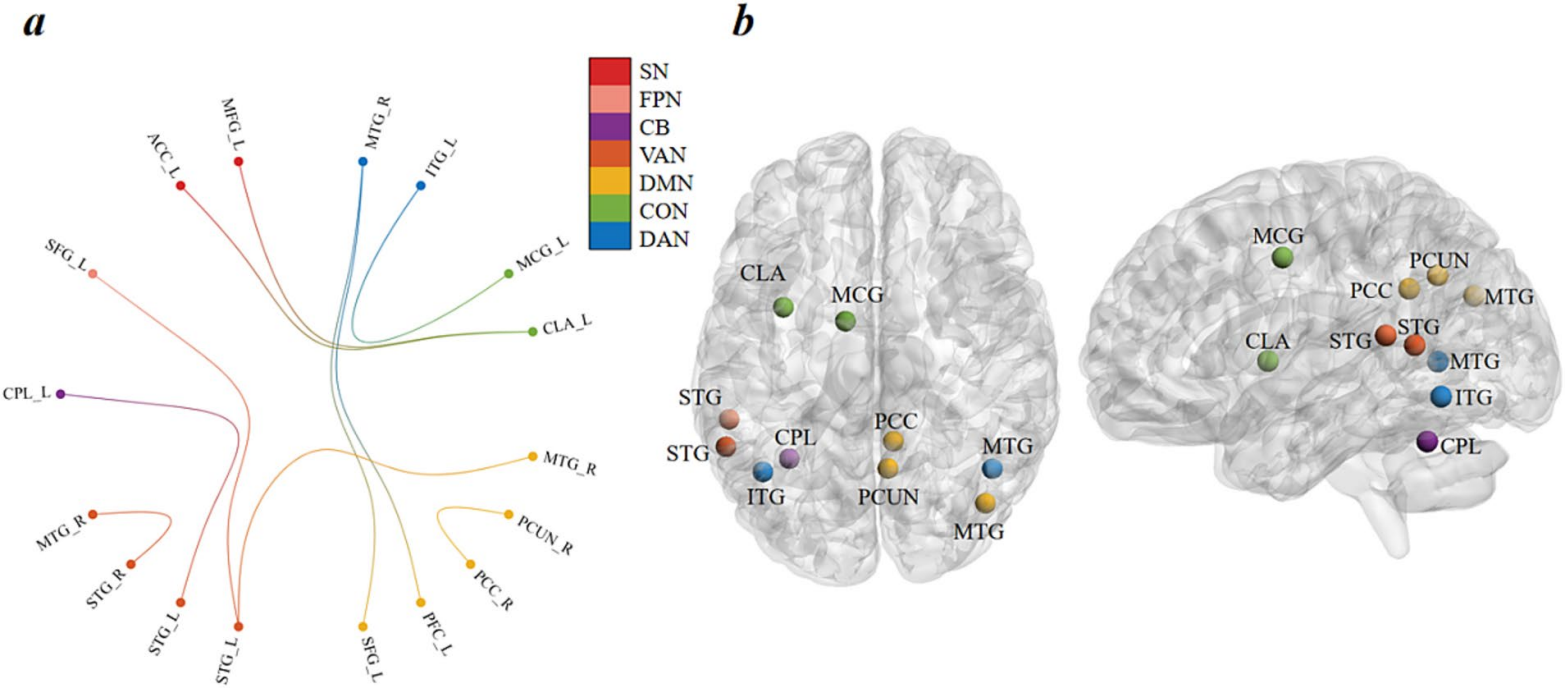
Visualization of all edges predicting individual depression. **(A)** The functional connections in positive networks, plotted as the number of connections within each lobe. MTG, middle temporal gyrus; ITG, inferior temporal gyrus; MCG, middle cingulate gyrus; CLA, claustrum; PCUN, precuneus; PCC, posterior cingulate cortex; PFC, prefrontal cortex; SFG, superior frontal gyrus; STG, superior temporal gyrus; CPL, cerebellum posterior lobe; ACC, anterior cingulate cortex; MFG, middle frontal gyrus. **(B)** The perspectives of the top 10 nodes related to depression.

CPM was applied to identify all potential edges related to depression, in order to discern neuromarkers of depression. First, we found a FC pattern significantly associated with depression, mainly including connections of DMN and VAN. And our findings revealed significant correlations between observed and predicted depression scores (r = 0.13, *p* < 0.005). Figure 1 displays the outcomes for various other CPM thresholds. Moreover, Figure 2A depicts the visualization of all depression network edges. Additionally, we feed the depression’s positive mask from CPM into GRETNA for calculating the degree center numbers of all mask nodes. Figure 2B illustrates the foremost 10 nodes in terms of degree centrality, whereas Table 2 enumerates the 10 nodes with the most significant contribution figures indicative of depression. The top 10 nodes are grouped into four areas characterized by the greatest quantity of connections, that would be the CLA (left claustrum), MTG (middle temporal gyrus) and ITG (left inferior temporal gyrus), left cerebellum posterior lobe (CPL), left middle cingulate gyrus (MCG), left superior temporal gyrus (STG), right precuneus (PCUN) and right posterior cingulate cortex (PCC).

**Table 2:** Nodes contributed to predicting depression.

Node	Node_Name	Network	X	Y	Z
57	CLA_L	CON	−34.37	3.29	4.19
236	STG_L	VAN	−56.47	−50.48	9.92
257	MTG_R	DAN	46.09	−58.93	3.93
51	MCG_L	CON	−10.48	−2.1	42.02
80	MTG_R	DMN	43.43	−72.21	28
89	PCUN_R	DMN	5.91	−58.82	35.45
92	PCC_R	DMN	7.94	−48.37	30.57
109	PFC_L	DMN	−3.06	44.41	−9.46
114	SFG_L	DMN	−20.16	63.65	19.39
178	SFG_L	FPN	−22.53	10.76	63.73
212	ACC_L	SN	−10.76	25.99	24.54
214	MFG_L	SN	−27.5	52.04	21.28
237	STG_L	VAN	−55.3	−39.89	13.51
238	STG_R	VAN	51.52	−32.52	7.55
239	MTG_R	VAN	51.28	−28.52	−4.3
244	CPL_L	CB	−32.12	−55.03	−25.22
262	ITG_L	DAN	−42.26	−60.12	−8.85

CLA, claustrum; STG, superior gyrus; MTG, middle temporal gyrus; MCG, middle cingulate gyrus; PCUN, precuneus; PCC, posterior cingulate cortex; PFC, prefrontal cortex; SFG, superior frontal gyrus; ACC, anterior cingulate cortex; MFG, middle frontal gyrus; CPL, cerebellum posterior lobe; ITG, cerebellum posterior lobe; DAN, dorsal attention network; CON, cingulo-opercular network; DMN, default mode network; VAN, ventral attention network; CB, cerebellar; FPN, fronto-parietal; SN, salience network.

Subsequently, the Spearman correlation method evaluated a link between the strength of brain FC and the educational attainment of parents. Specifically, we found that 1 edges (CPL-STG, r = −0.106, *p* < 0.05) were negatively associated with father’s education level, and 2 edges (MFG-CLA, r = −0.145, *p* < 0.05, CPL-STG, r = −0.109, *p* < 0.05) were negatively associated with mother’s education level.

### 3.3 Mediating results

**Figure 3:**
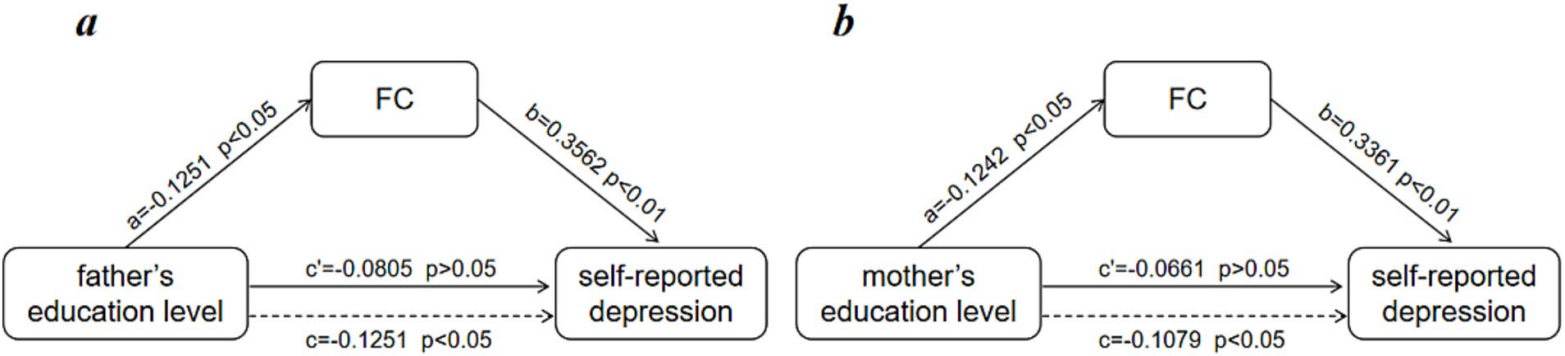
**(A)** Mediating effects of the brain FC related to depression on the relationship between father’s education level and depression. **(B)** Mediating effects of the brain FC related to depression on the relationship between mother’s education level and depression.

We employed a mediation analysis to investigate whether the brain FCacts as a bridge between parent’ s education levels and depression. Regressing parents’ education against brain depression-related measures, with SDS (Self-Rating Depression Scale) scores serving as the outcome variable, formed the study design. As shown in Figure 3A, mediation analyses indicated that the brain FC mediated the relationship between father’s education level and depression [β = −0.0835, 95% confidence interval (CI) = −0.0943 to −0.004, *p* < 0.05]. Moreover, Figure 3B showed that the brain FC mediated the relationship between mother’s education level and depression [β = −0.0702, 95% confidence interval (CI) = −0.086 to −0.0045, *p* < 0.05].

## 4 Discussion

This study used resting-state functional magnetic resonance imaging (rs-fMRI) to investigate the effect of parents’ SES as suggested by parents’ educational attainment, on depressive inclines. The probability of experiencing depressive symptoms decreases as parent’ s education levels increases. This study identified a FC pattern which can significantly predict invididual’s depressive symptoms, The most FCs within this pattern belong to DMN and VAN. Additionally, the FC pattern associated with depression serves as an intermediary in the interplay between parents’ SES and depressive symptoms.

The research demonstrated an inverse correlation between the depressive symptom and parents’ SES, consistent with earlier studies (Lorant et al., 2003; Elovainio et al., 2020; Whitfield et al., 2021). For example, parents with higher education typically engage in positive and rational parenting, which boosts adolescents’ mental well-being and thereby reduces the risk of depressive symptoms (Carr and Pike., 2012). The intergenerational impact of education suggests that parents with better education usually invest more time with their children, offering increased support and companionship, thereby promoting psychological well-being and reducing depressive tendencies (Oreopoulos et al., 2006). Furthermore, parents with higher education typically create an environment conducive to their children’s growth within the family, especially in terms of economics, opportunities, and resources (Reiss et al., 2019). In other words, higher parents’ SES is associated with a lower likelihood of developing depressive symptoms. Evidence also indicates that socio-economic struggles in early childhood are likely to lead to depression later in life (Luo and Waite, 2005; Mossakowski, 2008). Proponents of the accumulation risk theory contend that continuous or recurrent depression has a stronger link to parents’ SES than isolated episodes (Kendler and Gardner, 2010; Bromberger et al., 2011). Research indicates that among caregivers of elderly patients (usually adult children), there is a correlation between the socioeconomic status of the adult children and the physical health of the elderly patients (Giannouli and Tsolaki, 2022). Specifically, when caring for elderly patients, adult children may be influenced by their social class, as well as the role of economic capital, cultural health capital, and social capital, which in turn affects the physical health of the elderly patients (Simoni and Hilfiker, 2024). At the same time, as the condition of elderly patients deteriorates, the burden on their children increases due to the physical, psychological, and emotional stress of caring for the patient, which also has a certain negative impact on their mental and physical health (Sansoni et al., 2013). Moreover, the study confirms the differential effects of parents’ SES on various psychological variables throughout the lifespan. For instance, in older Greek adults, parents’ SES predicts spirituality and life satisfaction, with lower life satisfaction being associated with a higher tendency towards depression. However, the effect of parents’ SES on life satisfaction in young people is not significant (Giannoulis and Giannouli, 2020).

The research revealed that functional connectivity (FC) patterns linked to depression are predictive of depressive tendencies, consistent with earlier studies (Fitzgerald et al., 2021). For instance, research in the realms of structural and functional neuroimaging has revealed a correlation between the fundamental pathology associated with depression and the default mode network (DMN). The DMN, one of the principal functional connectivity networks activated while at rest, includes key regions such as the posterior cingulate cortex (PCC), angular gyrus, medial prefrontal cortex (mPFC), and precuneus (Raichle et al., 2001). The posterior part of the DMN, including the PCC and precuneus, is associated with maladaptive rumination in individuals with depression during rest. The mPFC, another constituent of the DMN, is thought to contribute to emotion processing (D’Argembeau et al., 2005; Schneider et al., 2008). Some studies indicate atypical activation of the mPFC during relaxation and introspection in individuals with depression (Sheline et al., 2009; Greicius et al., 2009). Research shows a correlation between the management of negative emotions in depressive states and DMN engagement, which is associated with depressive symptoms. Other research suggests that the DMN is involved in self-reflection and memory recall (Mak et al., 2017), and disorders of the DMN have been observed in patients with mental illnesses, including severe depression (Yan et al., 2019). Furthermore, impaired function of the attention networks, as indicated by prior research, is linked to psychological issues like depression due to its significant correlation with disrupted connectivity between the ventral attention network (VAN) and the dorsal attention network (DAN), impacting social, cognitive, and attentional functioning (Wainberg et al., 2022). Additionally, investigations using resting-state functional MRI (rs-fMRI) have revealed an association between depression and diminished capabilities of the VAN. A comprehensive meta-analysis reveals reduced foundational functional connections between VAN seeds and the precuneus in Major Depressive Disorder (MDD), affecting both the parietal and posterior cingulate cortices as well (Kaiser et al., 2015).

More and more evidence suggests that fMRI provides a non-invasive means to explore brain function, and functional connectivity (FC) in the resting state is based on observing slow, correlated fluctuations in brain regions during rest (Fox and Raichle, 2007; Biswal et al., 1995). Research has shown that parents’ SES is associated with the functioning of specific brain networks during development, such as the DMN and the VAN (Spreng et al., 2009; Seeley et al., 2009). Research indicates a correlation between increased maternal education with reduced network connectivity with external regions in the DMN, suggesting that higher maternal education corresponds to better network maturity (i.e., lower external functional connectivity; Chajes et al., 2022). Parent’ s education levels has a significant predictive value for family income (Gina et al., 2011). Families with lower parental education may have less favorable household incomes, and children from low-income families show reduced functional connectivity in the DMN (Tomasi and Volkow, 2023). Furthermore, this study also found that parents’ SES may impact the VAN, consistent with previous research results. For example, parental education significantly influences parent–child interaction, affecting cognitive and language development in children (Duncan and Magnuson, 2012). Therefore, parental education is associated with connectivity to auditory networks and systems related to executive and cognitive functions (e.g., VAN) (DeJoseph et al., 2022).

Additionally, this study’s mediation analysis reveals that the FC pattern linked to depression acts as a mediator between parents’ SES and depressive tendencies. For instance, within households of low socioeconomic status, children face increased challenges, specifically threats and deprivation, which act as mediators between their family’s economic standing and the emergence of depressive symptoms. Prolonged exposure to threats, rather than deprivation, correlates with heightened neural activity in the dmPFC, a central node of the DMN (Weissman et al., 2022). Furthermore, parents’ SES, particularly family income, and the association with trait depression are intermediated by the mPFC region, including its connectivity with the ACC volume and within the DMN. Additionally, research suggests that the relationship between parents’ SES and depressive symptoms may be influenced by parents’ SES as a proxy for early stress, adversity, and trauma (Yaple and Yu, 2019). Numerous studies suggest a connection between stress experienced in early life and alterations in the amygdala-ventral medial prefrontal cortex, along with pregenual anterior cingulate connections (Fan et al., 2014; Burghy et al., 2012), as well as other regions (Grant et al., 2015). These crucial connections, believed to be essential for efficient emotion management, are affected. Changes in the brain’s connections are especially noticeable in the region between the ventral or dorsolateral prefrontal cortex and the hippocampus among depressed individuals (Luking et al., 2011; Veer et al., 2010). Thus, the depression-related FC pattern mediates the relationship between parents’ SES and depressive symptoms.

## 5 Limitation

Future research should address the limitations of our study. First, Participants were asked to fill out the SDS, past experiences were measured by self-reporting, and the retrospective manner study design might limit the accuracy of the answers. Second, in this study, the cross-sectional data limits our exploration of the causal relationships between variables. Therefore, future research should collect longitudinal developmental data to elucidate the impact of parents’ SES on individual depressive emotions and its underlying neural mechanisms. Third, this study’s sample solely comprised college students, potentially restricting the study’s conclusion’s applicability. Future research may enhance study’s external validity by recruiting participants from a broader age range and cultural backgrounds.

## Data Availability

The original contributions presented in the study are included in the article/supplementary material, further inquiries can be directed to the corresponding author.
